# Neutrophil Gelatinase–Associated Lipocalin Acts as a Robust Early Diagnostic Marker for Renal Replacement Therapy in Patients with Russell’s Viper Bite–Induced Acute Kidney Injuries

**DOI:** 10.3390/toxins13110797

**Published:** 2021-11-12

**Authors:** Subramanian Senthilkumaran, Ketan Patel, Anika Salim, Pradeep Vijayakumar, Harry F. Williams, Rajendran Vaiyapuri, Ravi Savania, Namasivayam Elangovan, Ponniah Thirumalaikolundusubramanian, M. Fazil Baksh, Sakthivel Vaiyapuri

**Affiliations:** 1 Department of Biotechnology, School of Biosciences, Periyar University, Salem 636011, Tamil Nadu, India; maniansenthil76@gmail.com (S.S.); elangovannn@gmail.com (N.E.); 2 Emergency Department, Manian Medical Centre, Erode 638001, Tamil Nadu, India; 3 School of Biological Sciences, University of Reading, Reading RG6 6UB, UK; ketan.patel@reading.ac.uk; 4 School of Pharmacy, University of Reading, Reading RG6 6UB, UK; anika.salim@pgr.reading.ac.uk (A.S.); pradeep.vijayakumar@pgr.reading.ac.uk (P.V.); r.savania@reading.ac.uk (R.S.); 5 Research and Development Department, Toxiven Biotech Private Limited, Coimbatore 641042, Tamil Nadu, India; harry@toxiven.com (H.F.W.); raj@toxiven.com (R.V.); 6 Research Department, Trichy SRM Medical College Hospital & Research Centre, Trichy 621105, Tamil Nadu, India; ponniah.tks@gmail.com; 7 Department of Mathematics and Statistics, University of Reading, Reading RG6 6UR, UK; m.f.baksh@reading.ac.uk

**Keywords:** snakebite envenomation, Russell’s viper, viper bites, acute kidney injury, renal biomarker, neutrophil gelatinase–associated lipocalin, NGAL, renal replacement therapy

## Abstract

Snakebite-induced acute kidney injury (AKI) is frequently observed in patients following bites from vipers such as Russell’s viper (*Daboia russelii*) in India. Currently, the levels of serum creatinine are mainly used as a marker to determine the necessity for renal replacement therapy (RRT) (haemodialysis) in severe cases of AKI. However, it takes up to 48 h to ascertain a distinct change in creatinine levels compared to its baseline level upon admission. The time lost between admission and the 48 h timepoint significantly affects the clinical management of snakebite victims. Moreover, early diagnosis of AKI and decision on the necessity for RRT in snakebite victims is critical in saving lives, reducing long-term complications, and minimising treatment costs arising from expensive haemodialysis. Neutrophil gelatinase–associated lipocalin (NGAL) has been recently studied as a robust early marker for AKI in non-snakebite patients. However, its suitability for clinical use in snakebite victims has not been rigorously established. Here, we demonstrate the clinical significance of plasma NGAL as a robust marker for RRT following AKI using a large cohort (309) of Russell’s viper victims without any pre-existing health conditions. NGAL levels upon admission are positively correlated with creatinine levels at 48 h in different stages of AKI. Overall, NGAL acts as a robust early marker to ascertain the need for RRT following Russell’s viper bites. The quantification of NGAL can be recommended as a routine test in hospitals that treat snakebites to decide on RRT at early time points instead of waiting for 48 h to confirm the increase in creatinine levels. The diagnostic use of NGAL in Russell’s viper victims with pre-existing comorbidities and for other vipers should be evaluated in future studies.

## 1. Introduction

Snakebite envenomation (SBE) is a high priority, neglected tropical disease that predominantly affects rural communities living in developing countries such as India [1,2]. Among the Indian ‘Big Four’ snakes (Russell’s viper, Indian cobra, common krait, and saw-scaled viper), Russell’s viper (*Daboia russelii*) is responsible for most incidents and subsequent deaths and disabilities in rural India [3,4,5,6]. In addition to haemotoxic, neurotoxic, and myotoxic envenomation effects, nephrotoxicity in the form of acute kidney injury (AKI) in victims following Russell’s viper bites is frequently (around 15% of SBE victims [7]) observed [8,9]. AKI is also one of the key factors that contribute to SBE-induced deaths and long-term complications [10]. The severity of AKI following Russell’s viper bites varies widely depending on the amount of venom injected; locality of the offending snake; age, body mass, and existing health conditions of victims; and notably, the time delay between the bite and hospital treatment [11]. The proportion of patients with AKI (including non-SBE cases) requiring renal replacement therapy (RRT, which indicates the necessity for haemodialysis) varies from 25% to 100% [7,12,13]. In current clinical practice, the levels of serum creatinine, blood urea nitrogen, urinary albumin/proteins, and urine outputs are considered as biomarkers to determine the functional status of kidneys and severity of AKI [14]. However, these conventional markers are not ideal to establish the injuries arising from haemodynamic changes in kidneys that lead to variations in glomerular filtration rate, particularly during acute damage [14,15]. Moreover, serum creatinine and blood urea levels do not change promptly at the early phases of AKI, as the individuals with normal renal function have a functional reserve [16]. Thus, the glomerular filtration rate measured based on serum creatinine or other methods may not precisely reflect the early AKI [17]. It is also important to note that serum creatinine levels do not rise until over 50% of the renal glomeruli are affected. The elevation of serum creatinine appears from approximately 24 to 48 h following the renal injury/damage and thus, it does not aid in confirming SBE-induced AKI at early stages [15,16]. Generally, the serum creatinine levels are monitored for up to 48 h to confirm elevated levels before a decision is made regarding the need for RRT. Therefore, this type of clinical decision may be too late for SBE victims as the progression of pathological complications following SBE can be rapid. A robust early diagnostic tool to ascertain the necessity for RRT and subsequent timely intervention/treatment is likely to enhance the outcomes of SBE victims and reduce the substantial treatment costs arising from expensive haemodialysis.

Neutrophil gelatinase–associated lipocalin (NGAL), a 25 kDa protein belonging to the lipocalin family, has received significant attention in recent years as an early marker for AKI, as its levels are elevated in blood and urine much earlier than those of serum creatinine [18]. NGAL is expressed and released mainly from kidneys, neutrophils, epithelial cells, and the liver in response to various pathological conditions including AKI, inflammation, infection, and intoxication. The elevated NGAL level during AKI reflects structural injuries to the kidneys in contrast to serum creatinine, which demonstrates their functional status [18]. The clinical relevance of NGAL for SBE has not been explored until recently. In our preliminary studies, we reported the occurrence of elevated levels of NGAL following Russell’s viper bites long before the clinical manifestations and conventional serum creatinine levels [19,20,21]. Similarly, a few other studies have reported the prominence of plasma and urinary NGAL in SBE victims to determine the severity of AKI [9,22]. However, the clinical relevance of NGAL as a robust early biomarker to ascertain the need for RRT following Russell’s viper bites specifically in South India has not been effectively established. Hence, we have conducted a rigorous clinical study with a large cohort of Russell’s viper bite victims and vigorous exclusion and inclusion criteria and demonstrate the significance of plasma NGAL as a robust early biomarker for AKI to determine the need for RRT in these patients.

## 2. Results

### 2.1. Males and Working-Age Groups Are Largely Affected by Russell’s Viper Bites

**Figure 1:**
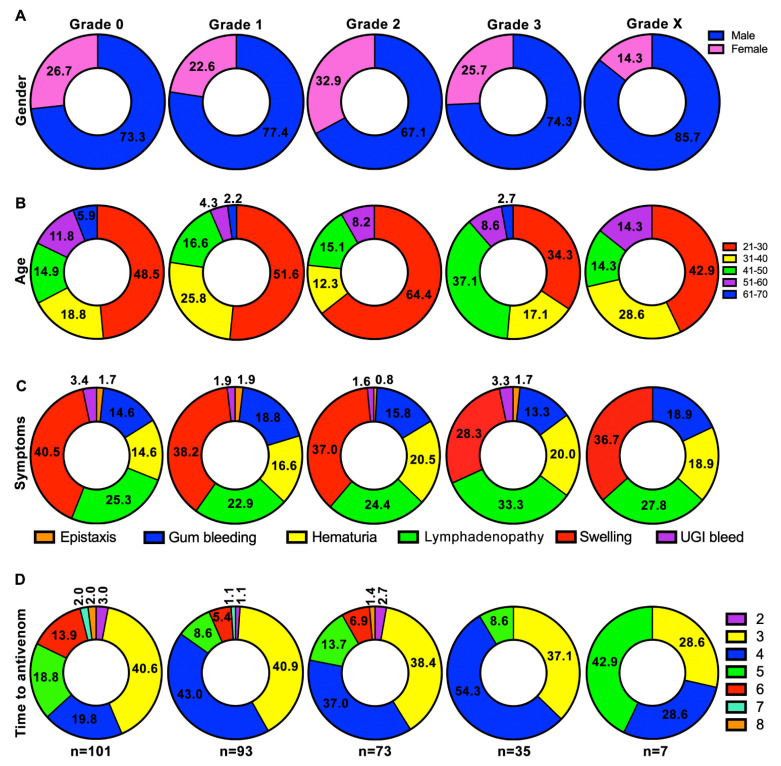
Characteristics of study population in different grades. (**A**) The distribution of males and females by grade. (**B**) Age distribution of patients in different grades. (**C**) Distribution of classical clinical symptoms of Russell’s viper bite in each category. (**D**) Distribution of elapsed time (hours) from bite to antivenom administration in different categories. This study only included patients who arrived at the hospital within 8 h following the bite. Multinomial logistic regression models were used to test whether gender, age, clinical symptoms, and time to antivenom were predictors of grade. The numbers shown on figure panels indicate the percentage in respective categories.

Patients who had pre-existing diabetes, hypertension, and renal diseases as well as other infectious diseases were not included in this study (more details are provided in methods section). Following our exclusion and inclusion criteria, totally, 309 patients were recruited in this study, and they included 227 (73.5%) males and 82 (26.5%) females. They were further assigned into appropriate grades [by following the criteria provided by the acute kidney injury network (AKIN)] based on their serum creatinine levels or its fold increase at 48 h from baseline level creatinine at admission: grade 0 (101 patients)—74 (73.3%) males and 27 (26.7%) females; grade 1 (93)—72 (77.4%) males and 21 (22.6%) females; grade 2 (73)—49 (67.1%) males and 24 (32.9%) females; grade 3 (35)—26 (74.3%) males and 9 (25.7%) females; grade X (7)—6 (85.7%) males and 1 (14.3%) female (Figure 1A). These data suggest that males were more affected by Russell’s viper bites than females in this study cohort.

To determine the specific age groups that were largely affected by Russell’s viper bites, the age of study participants was analysed. Their age ranged from 21 to 70 years with a mean and median of 34.88 (SD = 11.23) and 30 years (IQR: 26 to 42 years), respectively. As shown in Figure 1B, the patients between 21 and 50 years old were mainly affected by Russell’s viper bites in all grades. Specifically, the age group of 21–30 included 51.4% of total victims. These data confirm that the working-age groups of this cohort were largely affected by Russell’s viper bites.

The clinical symptoms of Russell’s viper bites were then analysed in these patients. Here, most patients in all grades displayed classical Russell’s viper bite symptoms of swelling, lymphadenopathy, haematuria, and bleeding around their gums (Figure 1C). A small number of patients developed epistaxis and upper gastrointestinal bleeding. There was no significant difference detected among any of these symptoms between the patients in various grades.

Delay in seeking antivenom treatment results in excessive skeletal muscle and kidney damage among SBE victims. In this study, although we recruited patients who arrived at the hospital within 8 h following bites, the arrival time was very similar (no significant difference) in all the grades (mean arrival time: grade 0 = 4.1 h; grade 1 = 3.8 h; grade 2 = 3.9 h; grade 3 = 3.7 h; grade X = 4.1 h) (Figure 1D). Based on the clinical symptoms and clotting parameters (specifically, prothrombin time/international normalised ratio of clotting test (PT/INR)), the patients received antivenom treatment. As shown in Figure 1D, all patients in this study received intravenous infusion of polyvalent antivenom raised against the Indian ‘Big Four’ snakes (Bharat Serums and Vaccines, India). The antivenom doses varied from 10 (100 mL) to 30 (300 mL) vials based on the severity of symptoms and clotting complications. The antivenom administration was started with 10 vials followed by monitoring of clotting parameters for every 6 h and additional infusion of 5–10 vials if PT/INR was still prolonged. In this study, none of the patients received more than 30 vials of antivenom.

The urine output in patients who were in grade 0 was normal (i.e., >100 mL/hour). However, the urine output was reduced in patients in other grades: grade 1: 50–60 mL/hour; grade 2: 30–40 mL/hour; grade 3: 20–25 mL/hour. All the patients in grade 3 were treated with 4 to 6 cycles of haemodialysis based on their creatinine levels at 48 h. The same number of cycles was used for patients in grade X based on their other critical conditions (as detailed in the methods section).

Together, these data confirm that gender, age, clinical symptoms, and time to antivenom were not significant predictors of grade classifications (X^2^ = 56.7859, df = 52, and *p* = 0.301) for AKI. These suggest that the severity of bite and elevation of creatinine levels were likely to be due to other factors such as the amount of venom injected in these patients.

### 2.2. Creatinine Levels Are Significantly Increased over Time in Grades 1–3

**Figure 2:**
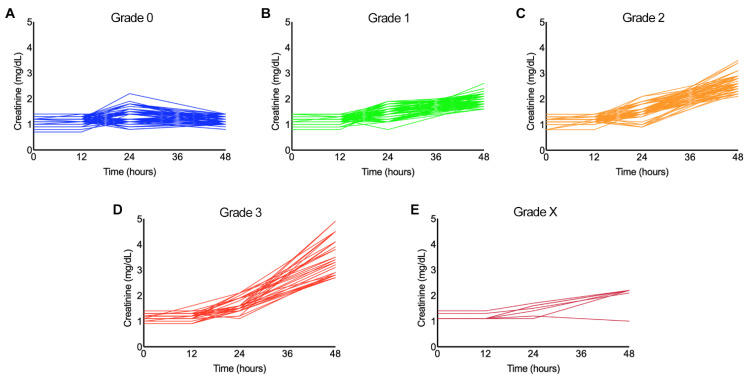
Patient profile plots of serum creatinine levels measured over 48 h in different grades. The levels of serum creatinine were measured at 0, 12, 24, and 48 h following admission for all patients in grade 0 (**A**), grade 1 (**B**), grade 2 (**C**), grade 3 (**D**), and grade X (**E**). For each category, we tested whether there is a significant change in mean creatinine level over time using generalised linear mixed effects models.

To determine the status of serum creatinine over time following the bite, its levels were measured at 0, 12, 24, and 48 h from admission. Patients’ profile plots of serum creatinine for different grades are provided in Figure 2. While there was no significant change (t = 0.870; *p* = 0.384) in the mean level of creatinine in grade 0 over the study period (Figure 2A), the mean rates of change in creatinine levels for all other grades were significantly different from that of grade 0 (*p* values < 2e^−16^ for grades 1–3 and X). The estimated mean increase in creatinine level in grade 1 was 0.273 mg/dL/day (SE = 0.0096) (Figure 2B), grade 2 was 0.468 mg/dL/day (SE = 0.0152) (Figure 2C), grade 3 was 0.634 mg/dL/day (SE = 0.0348) (Figure 2D), and grade X was 0.315 mg/dL/day (SE = 0.0310) (Figure 2E). These data suggest that the antivenom treatment did not completely prevent the progression of AKI (as measured via creatinine levels) and the necessity for haemodialysis among these patients, especially in grade 3 and X.

### 2.3. NGAL Acts as a Robust Biomarker for Grades 3 and X

**Figure 3:**
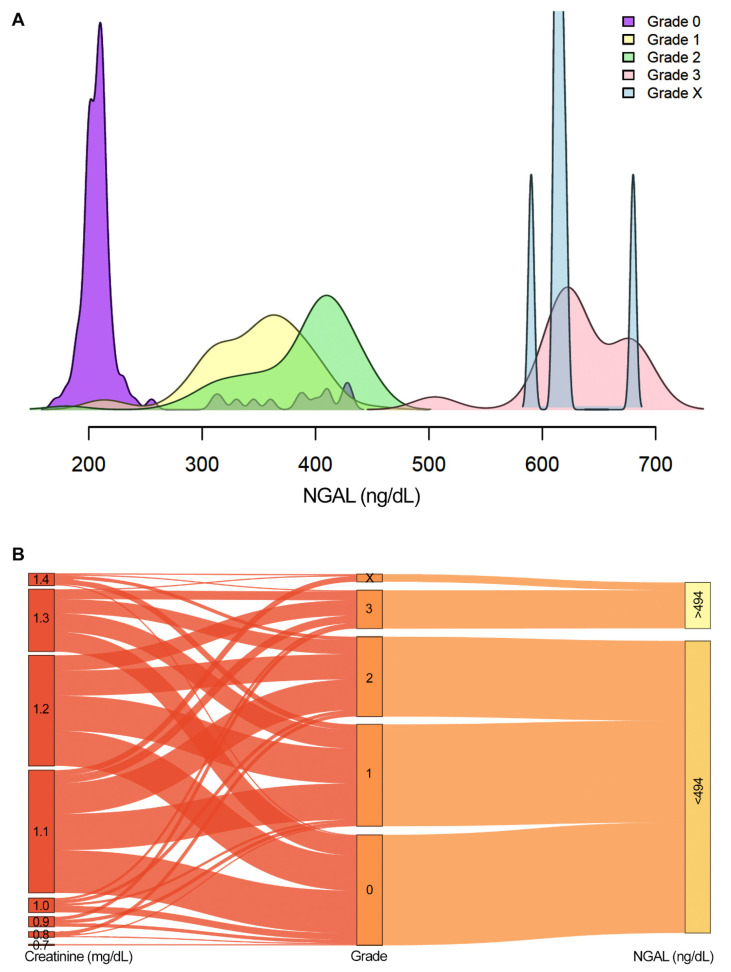
NGAL levels measured upon admission in different grades. (**A**) Smoothed density plots of the NGAL levels of the study population measured upon admission for different grades. Negligible overlap was observed in the NGAL values of grades 3 and X with other grades. (**B**) Sankey plot illustrates the association between serum creatinine levels at 0 h with NGAL levels measured at the same time point in different grades. All patients in grades 3 and X had more than 494 ng/dL of NGAL upon admission, while their creatinine levels at time 0 did not align with the grade classification at 48 h. The association between creatinine values at 48 h and NGAL was analysed using a linear model and adjusted for gender, age, clinical symptoms, and time to antivenom.

To determine whether NGAL could acts as an early diagnostic marker for Russell’s viper bite–induced AKI that necessitates RRT, its plasma level was measured in all the patients upon admission (i.e. 0 h). NGAL levels varied widely among different grades: grade 0—170 to 430 (mean 228.9; SD = 61.27); grade 1—210 to 450 (mean 346.7; SD = 45.63); grade 2—180 to 450 (mean 387.7; SD = 48.72); grade 3—500 to 700 (mean 633.7; SD = 43.40); grade X—590 to 680 (mean 620.6; SD = 27.91) (Figure 3A). NGAL was a highly significant predictor for creatinine levels at 48 h and positively correlated with the elevation of creatinine levels when adjusted for gender, age, symptoms, and time to antivenom (F = 540.6; df = 1, 282; *p* = 2.2e^−16^) (Figure 3B). However, NGAL levels were not significantly positively correlated with time to antivenom treatment. The Youden index was used to identify the best cut-off level for plasma NGAL to decide on the need for RRT in Russell’s viper bite victims. The mean value for this index based on 5000 bootstrap samples was found to be 493.75 ng/dL (SD = 20.02) with a sensitivity and specificity of 98% (SD = 0.03) and 100% (SD = 0.01), respectively, with an area under the curve (AUC) of 1.0. The accuracy was 99% (SD = 0.01). Hence, a cut-off value of >494 ng/dL of NGAL may act as a best indicator to ascertain the necessity for haemodialysis.

**Figure 4:**
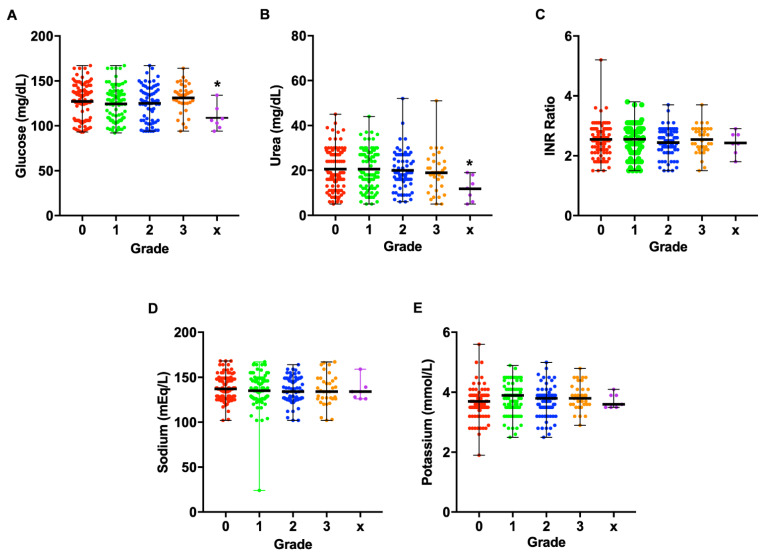
Distinct parameters relevant to AKI measured in different grades. Blood glucose (**A**), urea (**B**), INR ratio (**C**), sodium (**D**), and potassium (**E**) levels were measured in the study population among different grades at the time of admission. These values did not significantly differ between grades. There was no association between NGAL and these clinical parameters as analysed using a linear model, adjusted for gender, age, clinical symptoms, and time to antivenom.

Furthermore, the levels of blood glucose (Figure 4A), urea (Figure 4B), INR (Figure 4C), sodium (Figure 4D), and potassium (Figure 4E) did not significantly differ between grades. Similarly, they did not significantly associate with the levels of NGAL in all grades (*p* values of test for joint association with NGAL were as follows: grade 0: F = 1.7784, df = (5,82), *p* = 0.1264; grade 1: F = 0.3313, df = (5,74), *p* = 0.8925; grade 2: F = 1.4198, df = (5,55), *p* = 0.2316; grade 3: F = 0.0734, df = (5,16), *p* = 0.9955).

Overall, these data suggest that an NGAL value of above 494 ng/dL upon admission indicates the necessity for RRT among Russell’s viper bite victims with AKI. Hence, RRT can be initiated in these patients earlier instead of waiting for 48 h for creatinine levels to get increased as an indicator.

## 3. Discussion

SBE is a major public health issue in rural tropics as it causes a significant number of deaths, permanent disabilities, and economic loss among rural communities [2,4]. Notably, the World Health Organisation has classified SBE as a high-priority, neglected tropical disease with a strategic map that aims to reduce SBE-induced deaths and disabilities by half before 2030 [23]. SBE victims (especially following viper bites) frequently develop AKI due to the direct actions of venom toxins on kidney tubules, decreased renal, or intrarenal perfusion because of venom-induced hypotension, inflammation, and oedema in the tubulointerstitial region, nephropathy following extensive haemolysis and/or rhabdomyolysis, infection due to the bite, and reduced filtering capacity of the glomerulus due to venom-induced hypoperfusion [11]. Since this wide range of SBE-induced complications in kidneys develops within a short period, AKI acts as a critical factor for SBE-induced deaths and long-term complications such as chronic kidney disease, prehypertension, and hypertension [10]. To retrieve the normal functions of kidneys, RRT as well as other supportive treatment measures are being used for SBE victims based on the severity of damage. Notably, pre-existing health conditions such as diabetes, hypertension, acute/chronic renal diseases, and inflammation in SBE victims may significantly increase the susceptibility to develop AKI and the necessity for extensive RRT. Currently, serum creatinine levels are mainly used to ascertain the necessity for RRT in SBE victims [15]. In most cases, urine outputs are also considered when deciding on RRT, although this is not a useful factor in all cases. Serum creatinine levels represent the normal functions of kidneys by measuring the glomerular filtration rate, which conventionally reduces with age. An abnormal rate for certain age groups is most likely to indicate unusual kidney function [17]. However, measuring serum creatinine levels is time consuming, and the levels are affected by other factors such as diet, exercise, or reduced muscle mass, suggesting that it is not a powerful predictor for true reduction in glomerular filtration rate [24]. Notably, it takes up to 48 h to exhibit an elevated level following AKI, and therefore, it is not an ideal marker to detect early AKI, for example, in SBE. Hence, it is crucial to develop a reliable biomarker that can detect AKI at its early stage in SBE victims.

NGAL has been studied as a valuable diagnostic marker for AKI over the recent years [25,26]. Based on the nature of the damage, either plasma or urinary NGAL is being considered as a specific biomarker to detect AKI [27,28,29]. We have previously proposed NGAL as an early marker for Russell’s viper bite–induced AKI [19,20,21]. In this study, using a large cohort of Russell’s viper bite victims who arrived at the hospital within 8 h with normal serum creatinine levels, we demonstrate that NGAL acts as a robust diagnostic marker to ascertain the need for RRT among patients who were classified in grades 3 and X of AKI. The elevated level of NGAL was prominent at the time of admission, while it took up to 48 h for creatinine levels to increase. Previously, urinary NGAL has been identified as a useful marker for AKI among Russell’s viper bite victims in Sri Lanka [9]. Another study identified urinary NGAL as a good marker for *Bothrops* species–induced AKI although the authors concluded that serum creatinine is the best marker [22]. A range of studies has explored the use of NGAL as an early and effective biomarker for AKI although there are certain limitations in its use under diverse settings. Some studies have reported NGAL to be a definitive biomarker for AKI [30,31,32] while others have acknowledged the utility of NGAL but do not yet consider it to be a gold standard marker for early detection of AKI [33,34]. One of these studies has suggested that urinary NGAL is not a robust biomarker for AKI, although it used a smaller cohort of patients who were already critically ill with sepsis, and it did not measure plasma NGAL [33]. Similarly, another study has highlighted the different molecular forms of urinary and plasma NGAL, and therefore, the authors considered that this is not a perfect marker for early AKI detection [34]. A multicentre study with a large patient cohort confirmed the utility of NGAL values for detecting AKI [35]. The use of NGAL-deficient mice demonstrated the strong relationship between NGAL and progression of diabetic nephropathy and confirmed NGAL as a useful biomarker [36]. Based on the existing literature, it is apparent that the use of NGAL as an early diagnostic marker may vary depending on the nature of AKI and associated comorbidities. However, this present study (which included 309 patients) has confirmed NGAL as a perfect marker for early detection of AKI among Russell’s viper bite victims who did not have any previous history of medical conditions. Comorbidities such as chronic kidney disease [37,38], diabetes [39], hypertension, and other cardiovascular diseases [40] were reported to increase the levels of plasma/urinary NGAL.

Currently, there is no standard cut-off value for plasma NGAL to decide on RRT in patients following viper (including Russell’s viper) bites. Since plasma NGAL is being enthusiastically considered as a point of care test in various clinical settings, it is important to identify the best cut-off value to decide on RRT at least in the context of SBE. Here, we identified a plasma NGAL value of 494 ng/dL as the best cut-off value because of its high specificity and sensitivity among the study cohort. Although there was no clear defining range for NGAL values among different grades, the patients (in grades 0–2) with a plasma NGAL value of below 500 ng/mL did not require RRT while patients in grade 3 and X who displayed an NGAL value of over 500 ng/dL certainly needed RRT in this study. The patients in grade X required RRT due to critical conditions that they developed although their serum creatinine levels were not increased. This demonstrates the significance of NGAL for SBE victims even if they do not display an elevated value of serum creatinine until 48 h after the bite. In addition to NGAL and/or serum creatinine values, it is important for the clinicians to continuously monitor the clinical signs and symptoms as well as other biochemical parameters to ascertain the need for RRT in exceptional situations where NGAL may fail to detect early SBE-induced AKI. Although the time to antivenom administration did not vary significantly between the patients in different grades in this study, the early arrival to the hospital is highly recommended to receive the antivenom treatment, which prevents a range of venom-induced complications. Moreover, the progression of AKI in SBE victims also relies on the amount of venom injected (or the severity of bite), and toxins composition.

In this study, males were more affected by Russell’s viper bites than females and this could be related to increased farming and other outdoor activities for men as reported previously [5]. Although the time delay between the bite and treatment is a critical factor in augmenting SBE-induced complications, here, we did not see any difference in the severity of AKI based on the time to antivenom treatment within 8 h. Previous studies, which have analysed patients who arrived up to 72 h following the bite, have demonstrated the time delay as a critical factor in induing AKI following viper envenomation [7,13]. Moreover, there was no significant difference between the glucose, sodium, potassium, urea, and INR levels among different grades, although this might be different when measured at later time points. Early detection of AKI using NGAL as a biomarker will aid in initiating early RRT to prevent further progression of renal damage. Moreover, this will significantly reduce the cost of treatment arising from RRT (dialysis). There was no death found in our study despite many patients suffering severe AKI in grades 3 and X. Since, NGAL values were reported to increase in patients with other health conditions such as cancer and inflammatory diseases [41], the pre-existing comorbidities in SBE victims are likely to influence the levels of NGAL.

To the best of our knowledge, this is a large study wherein plasma NGAL values were determined for 309 Russell’s viper bite victims with robust inclusion and exclusion criteria. Based on this study, we propose plasma NGAL as a reliable biomarker to diagnose and triage AKI among Russell’s viper bite victims at the time of admission much before the elevation of serum creatinine levels. This will allow the clinicians to initiate appropriate care including RRT at the earliest to reduce extensive renal damage and associated deaths following envenomation. Notably, early intervention for AKI with RRT will significantly reduce the treatment costs. Further studies are required to confirm whether NGAL can be used as a biomarker for AKI developing from the bites of other snake species in different countries. It is also important to ascertain whether NGAL can be used as a marker for Russell’s viper bites from different regions within [42] and outside of India such as Myanmar and Sri Lanka as intra-specific and regional variations were found in their venom compositions, especially in specimens that were found outside of India [43,44]. The minimum cut-off values of plasma NGAL may also differ among different ethnic groups living in geographically diverse areas. Hence, further studies to ascertain these factors will facilitate the use of NGAL as a point of care test for worldwide use.

## 4. Methods

### 4.1. Study Design

This prospective study was carried out at the Emergency Department of Manian Medical Centre, Tamil Nadu (a large state in South India with a high burden of SBE) from June 2018 to May 2021. This study was approved by the Institutional Ethical Review Committee at Toxiven Biotech Private Limited (reference number: ICMR—Toxiven Ethics 2018–001/002), Tamil Nadu. Informed written consent was obtained from every participant before their enrolment in this study. In total, 309 victims who were confirmed as Russell’s viper bite victims (based on dead/live specimens brought to the hospital and/or classical clinical symptoms) were included in this study. All patients were above 20 years of age, and we did not receive any patients with the age of <20 years old who met the inclusion criteria. These patients were presented at the hospital within 8 h following the bite, and their serum creatinine level was less than 1.5 mg/dL upon admission. Patients with bites from species other than Russell’s viper were excluded from this study. Furthermore, patients whose serum creatinine level was more than 1.5 mg/dL upon admission or who had arrived at the hospital after 8 h following bites were excluded from this study. Similarly, the patients who had pre-existing renal diseases, diabetes, hypertension, sepsis or systemic infectious diseases, haemodynamic instability of any cause, exposure to nephrotoxic drugs/chemicals, or other biological toxins and injuries or other comorbidities with or without medications were excluded from this study.

### 4.2. Data Collection

All the patients who were included in this study were subjected to detailed clinical examination and basic laboratory investigation at the time of arrival to the emergency department. Blood samples collected upon admission were used to estimate the level of plasma NGAL using the standardised Triage^®^ NGAL tests (Biosite Inc., San Diego, CA, USA). The demographic (e.g., age and gender), clinical (time of bite to calculate the interval between bite and antivenom administration and clinical symptoms such as swelling, lymphadenopathy, bleeding in gums, epistaxis, upper gastrointestinal bleeding, and haematuria) and laboratory [e.g., the levels of serum creatinine, plasma NGAL, blood glucose, urea, sodium, potassium, and the international normalised ratio (INR) of blood clotting] data of all victims were collected and thoroughly verified by the authors prior to systematic recording of data for further analysis. All the patients were monitored hourly for urine output, and their serum creatinine levels were measured using the modified Jaffe method according to the manufacturer’s instructions at 0, 12, 24, and 48 h following admission. Patients were treated according to the standard protocols including for the antivenom administration and supportive measures. During their stay at the hospital, none of these patients developed hypotension, hypoxia, dehydration, or sepsis or received any nephrotoxic agents. Normal levels of serum creatinine and urine output were ensured prior to their discharge from the hospital.

### 4.3. Classification of Patient Groups

The grades of AKI were determined using serum creatinine levels at different time points based on the criteria provided by the acute kidney injury network (AKIN): grade 0 (or no AKI) had a serum creatinine level of <1.5 mg/dL or less than 1.5-fold increase at 48 h compared to their baseline level; grade 1—serum creatinine level of 1.5 to 2.1 mg/dL or 1.5- to 1.9-fold increase at 48 h; grade 2—serum creatinine level of 2.2 to 2.6 mg/dL or 2.0- to 2.9-fold increase at 48 h; grade 3—serum creatinine level of >2.6 mg/dL or >3-fold increase at 48 h. RRT was initiated in those patients with AKIN stage 3. A total of seven patients (grade X) received RRT based on their critical conditions instead of serum creatinine levels: three of them displayed volume overload due to hugely reduced or no urine output, two patients had severe acidosis, one suffered anuria, and another had severe rhabdomyolysis.

### 4.4. Statistical Analysis

Multinomial logistic regression models were used in the hypothesis test of whether gender, age, clinical symptoms, and time to antivenom are predictors of grade, and generalised linear mixed effects models were used to model change in creatinine levels over time. All statistical analyses were performed using the R statistical package (Version 3.6.2, R Foundation for Statistical Computing, Vienna, Austria) and GraphPad Prism (Version 7, GraphPad Software, San Diego, CA, USA).

## Data Availability

All data associated with this study are provided in this article.
